# Is Social Distancing Law the New Normal? Forced Shift to Media Online Learning and Its Effectiveness: A Moderating Role of Student Engagement During the Pandemic of COVID-19

**DOI:** 10.3389/fpsyg.2022.923996

**Published:** 2022-06-17

**Authors:** Qing Liu, Shuwen Mo

**Affiliations:** ^1^Hunan International Economics University, Changsha, China; ^2^Zhongkai University of Agriculture and Engineering, Guangzhou, China

**Keywords:** force-shift to media online learning, lack of social interaction, perception of maintaining social distancing and law, perceived harm, effectiveness of media online learning

## Abstract

The author intends to investigate the role of social distancing laws in the new normal as well as the effectiveness of forced shift to media online learning. This research indicates that student involvement had a moderating influence during the epidemic. This study is based on social learning theory (SLT), which endeavors to emulate the behavior, perceptions, and emotions of other individuals. The data were obtained from various Chinese universities. We gathered data utilizing the stratified sample approach as well as Google Form. A total of 256 students enrolled in a variety of programs at Chinese universities completed a questionnaire for this investigation. The direct, mediating, and moderating effects of the variables were evaluated using partial least square structural equation modeling in this study (PLS-SEM), using the Smart-PLS software 3.0. According to the findings, forced shift to media online learning acts as a mediator between the lack of social interaction, perceived harm, perception of maintaining social distancing, and the law, and the effectiveness of media online learning has a significant effect on the effectiveness of media online learning. In addition, student engagement appears to be a moderator between the forced shift to media online learning and its effectiveness. A future study might concentrate on gaining a deeper understanding of the numerous online engagement-promoting behaviors. Teachers must go above and above to provide themes that enable pupils to connect while learning. Instructors and educational institutions will benefit from the research since it will allow them to define methods for boosting social interaction in online learning and analyze methods for enhancing the efficacy of media in online learning.

## Introduction

There has been a horrific pandemic that has spread rapidly over the world since December 2019. The present outbreak is a new viral infection. Online media learning is a critical concern for all educational institutions. Because of media technology, students may engage in learning activities or what is known as an educational environment from any location (Mustafa et al., 2020). With the fast technological breakthroughs of the twenty-first century, it should be able to combine face-to-face learning with the use of technology to create a more effective learning environment (Ijaz Hussain et al., 2020). Physical education has given way to distance learning and, most recently, online education, in which students are taught in “unreachable classrooms” (Abou-Khalil et al., 2021). To maintain social distance and combat the transmission of the coronavirus, all public and private sectors have switched to online learning. Institutes have taken a step toward online learning, but that is not the best educational style. According to Mehall (2020), the lack of social interactions accounted for 31% of the differences in hurdles to online learning achievement. The findings suggest that a lack of social interaction is one of the barriers to successful online education, but that a lack of social interaction is not a barrier to successful online education during the pandemic if students’ attitudes toward preserving social distance and the law are controlled (Ndzinisa and Dlamini, 2022). Online education is a type of distance learning that helps students who are unable to attend traditional face-to-face classroom training to improve their educational outcomes. Teaching and learning procedures aided by ICT are effective choices for making their knowledge correspond with current technology developments and globalization, minimizing teaching errors, improving learning quality, and improving student understanding (Moorhouse, 2020). The effectiveness of media online learning has proven to be highly advantageous for self-regulated pupils.

The fast emergence of new technologies provides the context for the development of the mixed learning environment as a novel answer to contemporary concerns. The worldwide online learning market is predicted to increase by $93.64 billion between 2020 and 2024 (Miller, 2019). The worldwide economy is predicted to reach $336.98 billion by 2026, with an annual growth rate (AGR) of 9.1% from 2018 to 2026, thanks to a substantial shift in traditional educational styles. College and university closures will affect 1.5 billion young people from 188 countries by 11 May 2020, accounting for 72% of the world’s population, and the numbers are expanding by the day (Miller, 2019). According to Fatima (2022), the goal of educational performance in the virtual community is to be at least similar to learning offered through the center’s other delivery methods, particularly face-to-face and physical education.

Concerns have been raised about the value of education, its efficacy, learning results, and student happiness as a result of the epidemic’s forcible shift to media-based online learning. Online education has provided instructors with additional time for lesson preparation. However, rapid contact between teachers and students is lacking (Sekulic et al., 2020). Because the epidemic has resulted in a quick shift in online education, melancholy moods among students must be addressed for online education to be effective. Due to the breakdown and forced shift to online learning in public institutions, the effectiveness of media online learning is one of the primary concerns with universities in China. Because private universities monitor their students’ and teachers’ everyday activities, the effectiveness of media online learning is harmed more in the public sector (Latheef et al., 2021).

This research was done to determine why the efficacy of media-based online learning affects the learning outcomes of students who are required to transfer to online learning. An extensive study has been conducted on the link between the efficacy of media online learning and one’s power, ego, temperament, intelligence, moral fortitude, and the skills needed by one’s community, region, and government (Galanti et al., 2020). Wang et al. (2020) proposed the same thing about the concept of media, but it distinguishes between “media” and “learning” based on reason, claiming that the phrase “media in learning” means essentially an independent agency or emergence, while the term “learning” is construed as a situation that was formed to compel someone to engage in learning actions. The role of the media as a vehicle for channeling communication signals and learning to educate people is emphasized through online media (Rasmitadila et al., 2020). The online learning system is new to both teachers and students. They are not familiar with the most effective and efficient techniques of online teaching and learning. Many forms of research have yet to be undertaken to investigate the significance of the online learning system, but some have highlighted the efficiency of online learning (Cruwys et al., 2021).

Another reason is the belief that maintaining social distance and the law is important, as well as rules at universities that have worked to increase teacher–student contact in a variety of learning environments and maintain social distance through online learning. People’s lives are saved, and knowledge is accessible to develop strategies to prevent infection transmission. It Justified the economic importance of social distance during the coronavirus collapse (Luo et al., 2020). The authorities were granted rights to implement this policy, such as separating crowds in playgrounds, guaranteeing security in crowded stores, or breaking up a home celebration, including the ability to fine those who broke the guidelines (Wigg et al., 2020). It also shows how the teacher’s efficacy in online learning is harmed by a lack of social interaction. Social interaction in the classroom, class participation, instructional materials, teaching methods, and encouragement all have a positive impact on some factors such as learning outcomes, results, and fulfillment (Mehall, 2020). Another intriguing argument addresses the issue of perceived harm. Researchers looked into the effectiveness of media online learning and its impact on student satisfaction while adjusting for perceived harm and found it to be largely insignificant. Several research studies suggested that psychological health, job arrangements, mental health, stress, sadness, anxiety, and income were all affected by gender, demographic, and academic differences (Gautam and Gautam, 2021; Hills and Eraso, 2021). While there are various benefits to online learning, the lack of meaningful touch when studying online can hurt some learning areas, as cognitive and personal aspects of education are equally as important as precise material (Ölcer et al., 2020). Students’ digital literacy, gadgets, and time spent on online learning are some of the elements that have been included in various research. Students’ digital literacy, gadgets, and time spent on online learning are some of the elements that have been included in various research. Digital literacy and its effects on parents, teachers, and students point to several things, such as creating learning communities, getting the right information at the right time, and using the right order to get the right information (Kahu and Nelson, 2018; Baber, 2020).

This study deals with social distancing law the new normal, the forced shift to media online learning, and its effectiveness and the moderating role of student engagement. As a result, these ideas serve as a basis for the suggested theoretical framework, which is being tested experimentally in this study. The purpose of this research is to highlight the following study objectives to identify and achieve them as follows:

1. To examine whether perceptions of maintaining social distancing and the law, a lack of social interaction, and perceived harm have a significant effect on the effectiveness of media online learning.
2. To examine whether a forced shift to media online learning mediates the relationship between perception of maintaining social distancing and the law, lack of social interaction, perceived harm, and effectiveness of media online learning.
3. To examine whether students’ engagement moderates the relationships between the forced shift to media online learning and the effectiveness of media online learning.

## Review of Literature and Hypothesis Development

This framework is based on one important theory that analyzes the relationships between the perception of maintaining social distancing and the law, a lack of social interaction, perceived harm, and effectiveness of media online learning and forced shift to media online learning.

### Effectiveness of Media Online Learning

The effectiveness of media online learning is defined as “media technology that also brings learners into learning activities anywhere, or what is so-called the ubiquitous learning environment” (Prasasti et al., 2019). Teachers and students gain from the use of digital media because they can access learning materials and interact directly in the classroom as well as beyond the classroom through online media. Online education allows students to learn from anywhere and at any time while keeping social distance and fellow laws in mind (Brasoveanu et al., 2020). It is a costly and self-motivated type of education. It is ideal for students all over the world since it can be done at any time and from any location, which is why it is also known as flexible learning. Online learning is advantageous in that it allows us to learn anywhere and at any time, yet it is costly and lacks sufficient access in distant locations. To maintain the social distance between students and professors, all institutes must move their studies to online learning. You can learn whatever you choose; self-placed learning, improved communication and collaboration, self-motivation, and discovering whatever you need are just a few of the benefits of online learning (Baber, 2021). Students can build links between curriculum materials and framework and the devices they use every day by exposing them to smartphone platforms in laptop learning. Blended learning is the process of combining two or more instructional methods or methodologies to get the desired results. This technique allows students to use online learning materials, particularly those based on the web/blog, without having to leave the classroom (Abou-Khalil et al., 2021).

### Perception of Maintaining Social Distancing and Law

Social distance is defined as “the degree of acceptance or rejection of social interaction between individuals, especially those belonging to different social groups” (Kumar et al., 2021). To maintain social distance and fellow laws, all institutes have switched from offline to online education. According to online teaching and learning, the transmission of the virus is delayed by preserving social distance. Because all schools, universities, and colleges are moving toward online education, online learning is becoming more popular (Mehall, 2020). Because students and teachers in offline learning do not maintain a social distance, the virus spreads to everyone. Social distancing, defined as a space of at least 6 feet or two arms between oneself and other people when outside one’s own home, is a major government rule (Qian and Jiang, 2020). The authorities were granted rights to implement this policy, such as separating crowds in playgrounds, guaranteeing security in crowded stores, or breaking up a home celebration, including the ability to fine those who broke the guidelines. Fear of transmission causes students to avoid social events, limiting the type of social distance possible (Hills and Eraso, 2021). Some laws and rules decided by the government are as follows: 1. “Stay away from everyone! 6 feet away!” 2. “Wash your hands with soap for 20 seconds frequently!” 3. “Stop touching your face! Wear gloves to remind you!” (Ölcer et al., 2020; Hills and Eraso, 2021).

### Lack of Social Interaction

In online education, social interaction is the most significant factor in determining the outcome, motivation, and happiness of both the teacher and the students. “Social interaction” is described as “the mutual or reciprocal impact, which results in behavior modification, exerted through social contact and communication, which is established by inter-stimulation and reaction” (Moorhouse, 2020). As the outbreak and social separation continue, social and emotional tensions between close relatives and the community are forming (Farrell and Brunton, 2020). Several researchers have revealed the occurrence of self-healing diseases such as worry, tension, exhaustion, and paranoia in the face of pandemics, indicating cultural mental disorder (Rasmitadila et al., 2020). While social isolation and confinement in houses were vital during the epidemic, they greatly disturbed most families’ daily routines (Wijayanti and Pramono, 2020).

### Perceived Harm

Another element that influences the shift to online learning is perceived harm. A feeling of unease is elicited in situations such as virus infections to keep people away from danger and dangerous activities (Wong et al., 2021). Numerous studies have connected increased coronavirus fear to global health cooperation and engagement in preventative activities such as frequent cleaning. According to one poll, the phobia was also linked to a broader variety of social and economic preventive measures in response to vaccination programs (Kumar et al., 2021). Furthermore, because more people are pursuing online education, it is now exciting to explore the mediating influence of coronavirus infection on the link between online learning performance and student experience (Abou-Khalil et al., 2021). We want to look at the links between online learning materials, average e-learning performance, and students’ happiness from the perspective of the online learning platform in educational institutions, as well as the mediating influence of the student’s engagement. Schools and universities have been put on lockdown to prevent the virus from spreading further (Wang et al., 2020).

### Forced Shift to Media Online Learning

The coronavirus has had a major impact on education, teachers, and colleges all across the world, as well as a variety of other aspects of daily life. As a result of the disease outbreak, educational institutions, universities, and institutions all across the world were forced to seal their doors, allowing students to practice social exclusion (Caton et al., 2021). The above interpretation of learning media is based on the idea that the educational/learning process is the same as effective communication. There are several parts to communicating, including the “source of messages, messages, and recipients of messages, media, and feedback” (Ndzinisa and Dlamini, 2022). Furthermore, as no one knows when the global epidemic will reach its end, public institutions have chosen to generate instructional materials for students in all fields of study utilizing currently available technologies (Kumar et al., 2021). Most research companies focus more on the transfer of instructional content to the digital arena rather than specifically on online instruction and distribution methods because the rapid shift to online education is now a test of company readiness (Bond and Bedenlier, 2019). Learning media is everything that can be utilized to convey information from the source to the destination in such a way that it activates students’ ideas, emotions, attentiveness, and goals and desires to accomplish the educational targets (Ölcer et al., 2020).

### Student Engagement

Student engagement is generally acknowledged to be essential. Simply put, students who are engaged in their studies are more likely to succeed. On either hand, the mechanisms that lead to a pupil’s involvement are still to be identified, or the term “engagement” is employed in opposite ways (Caton et al., 2021). The social condition of a learner, which encompasses their behavioral and emotional patterns and gives a clear understanding of their education, is defined as engagement. Students’ engagement is crucial to their academic success and long-term retention (Kahu and Nelson, 2018). Successful student engagement techniques are never adopted as part of teachers’ pedagogical activities, despite providing a solid understanding of what the instructor should do to increase engagement. Using technology to increase student participation will result in a variety of intellectual and social results in the near and distant future. When student–teacher relationships are strong and students consider the teacher to be competent, supportive, and collaborative, that is, committed and effective, they are more able to intensify (Carter et al., 2020). Online teachers provide prompt, responsive, embedded assistance to their students, establishing their presence and effectively engaging students through structured and unstructured approaches. Confidence and satisfaction with learning, as well as interpersonal involvement in learning, are both affected by student personality (Abou-Khalil et al., 2021).

### Perception of Maintaining Social Distancing and Law and Effectiveness of Media Online Learning

Abou-Khalil et al. (2021) studied the primary aim of slowing the spread of the coronavirus. This necessitates maintaining social isolation; moreover, it keeps people away from others who are infected but asymptomatic in order to reduce harm (Qian and Jiang, 2020). For those who are not afflicted, social distancing and the law are compulsions that go against their ability to socialize. There are several positive benefits of social distancing and common law. However, due to decreased economic activity, social distancing imposes significant costs on society. Researchers looked into remote learning and found that online education requires social reinforcement, which is much more important when individuals are psychologically disconnected (Ölcer et al., 2020). Some laws and rules decided by the government are as follows: 1. “Stay away from everyone! 6 feet away!” 2. “Wash your hands with soap for 20 seconds frequently!” 3. “Stop touching your face! Wear gloves to remind you!” (Ölcer et al., 2020; Hills and Eraso, 2021).

As a result, to teach children while living at home, a regular online teaching-learning method was introduced. In reality, transitioning from a traditional learning system to a modern e-learning system and media online learning is difficult and will take time to become adopted and developed (Mustafa et al., 2020). Since this technology is new in poor countries, schools and universities may need to update their curricula to accommodate media online teaching and learning. Since this technology is new in poor countries, schools and universities may need to update their curricula to accommodate media online teaching and learning. WHO rules say that better learning outcomes can only be achieved by successfully distributing media online, which can only be done by giving teachers and students training in digital literacy (Baber, 2021). Therefore, H_1_ is proposed as follows:

H_1_: *Perception of maintaining social distancing and the law has a significant effect on the effectiveness of media online learning.*

### Social Interaction and Effectiveness of Media Online Learning

Latheef et al. (2021) have studied that online learning is as successful as conventional education, and the issues of academic dissatisfaction and loneliness must be addressed. Online education is ineffective and does not foster social interaction among learners, which is necessary for them to learn more effectively. Interaction has been identified as an essential component of both physical and digital educational environments in previous studies. The method of sharing awareness and facts with teachers and different students is known as interaction (Miller et al., 2019). In addition, Latheef et al. (2021) studied that the relationship between the teacher and the students has also been found to have a substantial impact on student satisfaction. In this study, it was discovered that social familiarity, as well as social contact, harms learners’ perceived learning success, which may be due to Korean conservative culture. Ijaz Hussain et al. (2020) studied that individual and professional level factors, such as closeness, have a significant effect on the learners’ learning outcomes at the start and during the online course. In this case, media online learning combines many tools, including resources (García-Morales et al., 2021). How well media training and learning activities work online depends on how many technologies are available and how well they work. Therefore, H_2_ is proposed as follows:

*H_2_: Social interaction has a significant effect on the effectiveness of media online learning.*

### Perceived Harm and Effectiveness of Media Online Learning

Abou-Khalil et al. (2021) investigated the various characteristics of media online learning networks and found that they had a beneficial impact on student achievement. The accessibility of an educational center is shown to be the most critical element in learner happiness. According to the principal regulator of coronavirus in everyday life, it is linked to higher levels of panic disorder. The mediating impact of promoting social separation diminishes the influence of social contact on the effectiveness of media online learning. Bond and Bedenlier (2019) investigated the performance of online learning and its effect on students’ satisfaction while controlling for presumed coronavirus danger and discovered it to be mostly negligible. The presumed danger of the disorder is observed to have a significant impact on public perceptions of online learning (Wijayanti and Pramono, 2020). Therefore, H_3_ is proposed as follows:

*H_3_: Perceived harm has a significant influence on the effectiveness of media online learning.*

### Forced Shift to Media Online Learning and the Effectiveness of Media Online Learning

Mehall (2020) has studied that the vast majority of academic institutions, particularly in advanced places of the world, lack the required information and communication technology infrastructure to effectively engage in media online teaching and learning. It necessitates the expertise and skills of teachers and lecturers not only just in terms of digital procedures but also in terms of the forms utilized for online courses (Moorhouse, 2020). Furthermore, most learners do not have access to online learning because they do not have computers or smartphones. Rasmitadila et al. (2020) have studied that some students are unfamiliar with media online learning. Furthermore, many teachers and lecturers, especially in different areas of China, are not yet proficient in using Internet technology. Increasing these factors has a good or detrimental effect on the ability to complete and the feasibility of online education, with architectural execution performing a key role in determining the effectiveness of media online learning. Media online learning is equipped with community collaboration features and other methods for uploading tasks, handling questionnaires, and administering tests. It seems to have become a necessary component of today’s school environment. Media online education offers greater access to the latest knowledge as well as opportunities for social engagement and cooperation (Baber, 2021). Therefore, it is hypothesized as follows:

*H_5_: Forced shift to media online learning has significantly influenced the effectiveness of media online learning.*

### Mediating Role of the Forced Shift to Media Online Learning

Many educational institutions have introduced online training and educational methods all over the world. The majority of institutions were unprepared for the unparalleled transmission of instruction through the Internet, and the majority of learners were psychologically and physically poorly prepared for the transition to online education (Zolnikov and Furio, 2021). Some instructors and teachers have never been specifically qualified to educate through the Internet, and students have no previous experience with online learning. As a result, the rapid and progressive shift to online education caught most institutions, lecturers, and students off guard. Rahman et al. (2021) have suggested that eligibility in online education has exploded in media online learning over the last two centuries since the media online learning style refers to a wide population of learners with a range of demands that traditional face-to-face sessions are unable to provide. For self-regulated learners, online learning has proven to be very successful. According to Wigg et al. (2020), “Media online learning is more self-guided.” With the advent of digital technology, schools and universities are modifying their training methods to fulfill the challenges of users to provide an appropriate platform. One of the advantages of online learning is versatility. Students can plan their studies whenever they want, and professors may reuse previously prepared course content (Losada-Baltar et al., 2021). For a media online learning platform to work well, different technical tools must work well, and students must know what, where, when, and how to use learning activities to get the most out of the process (Bond and Bedenlier, 2019). Therefore, H_4_ is proposed as follows:

H_4_: *Forced shift to media online learning mediates the relationship between perception of maintaining social distancing and law, social interaction, and perceived harm.*

### Moderating Role of Students Engagement

Caton et al. (2021) studied that there was a general drop in student interest in online classes. To engage students in online courses, instructors, instructional designers, and system designers must know which interaction techniques are most successful. Previous research aimed at extracting effective engagement strategies was mostly performed in developing countries and in online learning contexts that necessitated a lot of preparation (Abou-Khalil et al., 2021). Both online and offline, classmate communication is seen as a key component of student engagement (Kahu and Nelson, 2018). Students’ engagement can be increased by using community chats. A cooperative-based appropriate learning design has already been shown to improve students’ interests as well as their social participation in the course. Dhawan (2020) suggested that during online courses, placing students in small, permanent discussion groups will improve student–student interaction. Student involvement is commonly recognized to be important. Bluntly defined, students who are involved in their subjects are more able to win (Bond and Bedenlier, 2019). Therefore, it is hypothesized as follows:

*H_6_: Students’ engagement moderates a relationship between forced shift to media online learning and the effectiveness of media online learning.*

**FIGURE 1:**
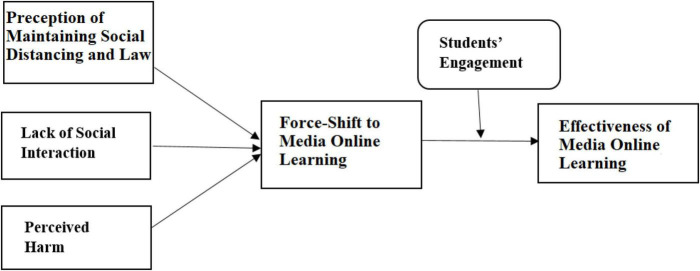
Conceptual framework.

Figure 1 has been formed based on the above literature and the hypothesis.

## Methodology

This study used a quantitative research methodology to test the hypothesis developed to analyze the influence of independent variables on the dependent variables. Using this strategy, bias has been decreased. This study population consisted of students enrolled in a range of programs at Chinese institutions. The study sample was selected using stratified sampling (Avotra et al., 2021; Yingfei et al., 2021; Nawaz et al., 2022). As a consequence, 256 questionnaires were distributed. In addition, the study employs a cross-sectional approach to learn about the participants’ perspectives on the perception of maintaining social distance and law, lack of social interaction and perceived harm in their institution, and students’ engagement according to the forced shift to media online learning toward institutions and the effectiveness of media online learning to a certain extent. The researcher created a five-point Likert scale for the survey instrument.

### Instrument for Data Collection

This study adapted the perception of maintaining social distance and the law scale with three items (Kleczkowski et al., 2015). The lack of social interaction was measured with a six-item scale by Kleczkowski et al. (2015). In this study, every item in this construct identifies the type of social interaction of the current students of the institutions. The perceived harm was measured with a seven-item scale by Zaman et al. (2021). One item is “I feel being at risk on campus for getting COVID-19.” The forced shift to media online learning was measured with a five-item scale by Zaman et al. (2021). Student engagement was measured with an eight-item scale. The effectiveness of media online learning was measured with a five-item scale by Kleczkowski et al. (2015).

### Demographics Details

Table 1 shows male respondents accounted for 65.4% of all responses, compared to 34.6% for female respondents. In Chinese culture, men hold a stronger role than women, particularly in universities. When it comes to qualifications, the majority of the respondents are doing a master’s degree (37.1%), then a bachelor’s degree (33.2%), and others (29.7%). Furthermore, it was shown that over 64% of the responses were from metropolitan areas, while the remaining 35% come from rural areas.

**TABLE 1:** Demographic analysis.

Demography	Description	Responses	%
Gender	Male	161	65.4
	Female	88	34.6
Qualification	Bachelors	85	33.2
	Masters	95	37.1
	Ph.D. or others	76	29.7
Residence	Rural	90	35.2
	Urban	161	64.8

*N = 256.*

## Data Analysis and Results

Smart-PLS 3.0 software was used to analyze the data. This program is commonly used to model structural equations (SEM) (Nawaz et al., 2019). Smart-PLS assists with data analysis in two stages. A measurement model is used in the initial stage to investigate the data validity and dependability (An et al., 2021). The validity of the data is tested using “factor loadings for each item, average variance extracted (AVE), hetero trait mono trait ratio (HTMT ratio), and Fornell and Larcker criterion”. In contrast, data suitability is determined using the “Cronbach alpha and composite reliabilities.” The study is then assessed using a structural model, in which the hypothesis is based on the values of “t-statistics, sig-values, the sample mean, and standard deviation.”

### Measurement Model

**FIGURE 2:**
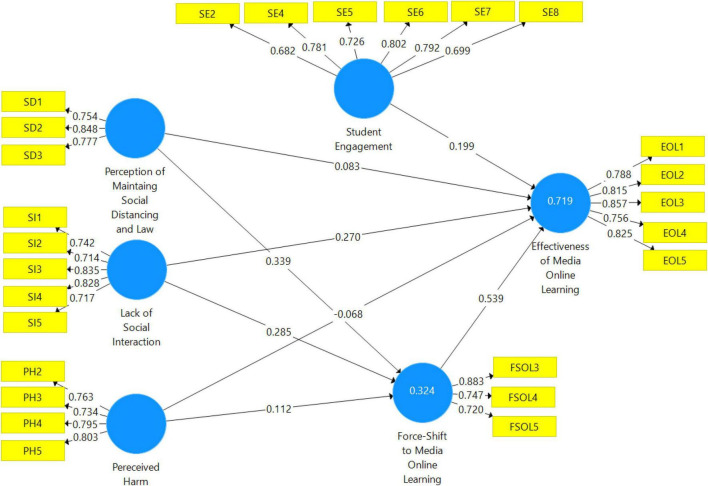
Output of measurement model algorithm.

The measurement model was used to ensure the “model’s validity and reliability” (see Figure 2). The “outer loading should be 0.5 and above,” and the “average variance extracted should be greater than 0.5,” according to their rule of thumb (Nawaz et al., 2020). According to the following argument, all items in the outer loading that are less than 0.5 should be eliminated one by one, starting with the lowest value.

It is recommended that the composite reliability be approved at a minimum of 0.70 and the AVE be at a minimum of 0.5 (Hair et al., 2020). A high level of reliability can be shown in Table 2 where all constructs have an AVE cutoff point of 0.50, a sign that the measurement model is reliable. For this investigation, Cronbach’s alpha was computed to determine the data’s internal consistency. Moreover, alpha is determined by the following rule: “α > 0.9 is excellent, α < 0.8 is good, and α < 0.7 is acceptable” (Xiaolong et al., 2021). All constructions’ Cronbach’s alpha, composite reliability, and AVE values are provided in Table 2.

**TABLE 2:** Factor loadings, reliabilities, and AVE.

Construct	Item	Loadings	CA	CR	AVE
Perception of	SD1	0.746	0.707	0.836	0.630
maintaining social	SD2	0.854			
distancing and law	SD3	0.777			
Lack of	SI1	0.751	0.826	0.878	0.591
social	SI2	0.723			
interaction	SI3	0.829			
	SI4	0.821			
	SI5	0.712			
Perceived	PH2	0.769	0.777	0.857	0.600
harm	PH3	0.728			
	PH4	0.789			
	PH5	0.808			
Force-shift to	FSOL3	0.883	0.703	0.828	0.618
media online	FSOL4	0.746			
learning	FSOL5	0.720			
Student	SE2	0.682	0.843	0.884	0.560
engagement	SE4	0.718			
	SE5	0.726			
	SE6	0.802			
	SE7	0.792			
	SE8	0.699			
Effectiveness of	EOL1	0.785	0.868	0.904	0.653
media	EOL2	0.813			
online	EOL3	0.866			
learning	EOL4	0.749			
	EOL5	0.824			
					

Table 2 shows that there is no issue with construct reliability, Cronbach’s alpha, composite reliability, and AVE of all the latent variables among the indicators of all constructs, except FSOL1, FSOL2, PH1, PH6, PH7, SE1, and SE3.

According to Table 3, the use of latent variables retrieved with a value of 0.50 or more is recommended for determining discriminant validity. Discriminant validity is indicated when the square root of AVE is greater than the value of latent variables.

**TABLE 3:** Fornell and Larcker criterion.

	EMOL	FSMOL	SI	SD	PH	SE
**EMOL**	**0.808**					
**FSMOL**	0.780	**0.786**				
**SI**	0.592	0.459	**0.769**			
**SDAL**	0.504	0.475	0.397	**0.794**		
**PH**	0.235	0.281	0.354	0.202	**0.774**	
**SE**	0.565	0.482	0.331	0.362	0.195	**0.748**

*EMOL, effectiveness of media online learning; FSMOL, forced shift to media online learning; SE, student engagement; SI, lack of social interaction; PH, perceived harm; SDAL, perception of maintaining social distancing and law.*

*Bold values show the relationship and significance.*

### Structural Model

**FIGURE 3:**
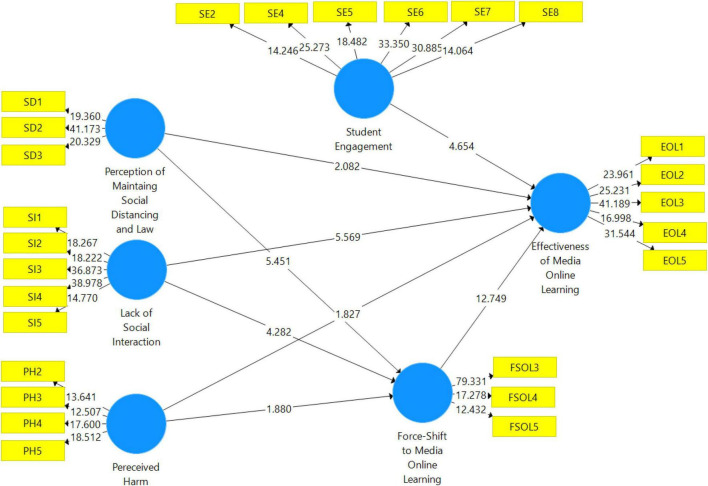
Output of structural model bootstrapping.

Three structural models (refer to Figure 3) were used in this study, namely, the direct-relation structural model, the mediation structural model, and the structural model that included moderating variables. There are three types of testing used in this study: hypothesis testing, mediator hypothesis testing, and moderator hypothesis testing.

In Table 4, in the study’s hypothesized model, a structural model is used to show the relationship’s reliance on one another. According to Smart-PLS 3.0, output findings are included in the table, including path coefficients, *t*-values, *p*-values, and standard errors (Hair et al., 2020). They were used by the researcher to determine whether the hypothesis was supported or not.

**TABLE 4:** Direct effects of the variables.

	*B*-value	Standard deviation	*T*-value	*p*-value	Decision
FSMOL – >EMOL	0.545	0.042	12.894	0.000	Accept
SI – >EMOL	0.286	0.046	5.841	0.000	Accept
SI – >FSMOL	0.286	0.062	4.609	0.000	Accept
PH – >EMOL	−0.067	0.039	1.752	0.000	Accept
PH – >FSMOL	0.118	0.057	1.967	0.025	Accept
SDAL – >EMOL	0.079	0.037	2.229	0.013	Accept
SDAL – >FSMOL	0.339	0.060	5.655	0.000	Accept
SE – >EMOL	0.198	0.042	4.717	0.000	Accept

*EMOL, effectiveness of media online learning; FSMOL, forced shift to media online learning; SE, student engagement; SI, lack of social interaction; PH, perceived harm; SDAL, perception of maintaining social distancing and law.*

The study’s first hypothesis (H1) states that a forced shift to media online learning impacts the effectiveness of media online learning. The result of this hypothesis shows that the t-statistics is 12.89 with a *p*-value of 0.000, thus this hypothesis has been accepted. The study’s second hypothesis (H2) states that social interaction impacts the effectiveness of media online learning. The result of this hypothesis shows that t-statistics is 5.84 with a *p*-value of 0.000, thus this hypothesis has been accepted. The study’s third hypothesis (H3) states that social interaction impacts the forced shift to media online learning. The result of this hypothesis shows that t-statistics is 4.609 with a *p*-value of 0.000, thus this hypothesis has been accepted. The study’s fourth hypothesis (H4) states that perceived harm impacts the effectiveness of media online learning. The result of this hypothesis shows that t-statistics is 1.752 with a *p*-value of 0.000, thus this hypothesis has been accepted. The study’s fifth hypothesis (H5) states that perceived harm impacts forced shift to media online learning. The result of this hypothesis shows that t-statistics is 1.967 with a *p*-value of 0.025, thus this hypothesis has been accepted. The study’s sixth hypothesis (H6) states that social distancing and law harm impact the effectiveness of media online learning. The result of this hypothesis shows that the t-statistics is 2.229 with a *p*-value of 0.013, thus this hypothesis has been accepted. The study’s seventh hypothesis (H7) states that social distancing and law impact forced shift to media online learning. The result of this hypothesis shows that t-statistics is 5.655 with a *p*-value of 0.000, thus this hypothesis has been accepted. The study’s eighth hypothesis (H8) states that student engagement impacts the effectiveness of media online learning. The result of this hypothesis shows that the t-statistics is 4.717 with a *p*-value of 0.013, thus this hypothesis has been accepted.

The purpose of the mediation test was to determine which mediating variables increased the impact of the independent variable on the dependent variable. As for the current investigation, the researcher used the resampling mediation technique (bootstrapping) to examine the indirect influence of each prospective variable (refer to Table 5).

**TABLE 5:** Mediator hypothesis testing.

	*B*-value	(STDEV)	*T*-value	*p*-value
FSMOL*SE – >EMOL	−0.074	0.030	2.384	0.009

*EMOL, effectiveness of media online learning; FSMOL, forced shift to media online learning; SE, student engagement.*

* shows relationship impact of two factors has been checked on another variable.

A test of moderation (see Table 6) was conducted to determine which moderator variable influences the direction or intensity of the association between the independent and dependent variables. Table 6 shows that student engagement (*B* = -0.074, *p* = 0.009) moderates the relationship between forced shift to media online learning and the effectiveness of media online learning, so this hypothesis is accepted.

**TABLE 6:** Moderator hypothesis testing.

	*B*-value	Standard deviation	*T*-value	*p*-value	Decision
PH- > FSMOL- > EMOL	0.600	0.031	1.940	0.26	Accept
SDAL- > FSMOL- > EMOL	0.183	0.035	5.156	0.000	Accept
SI- > FSMOL- > EMOL	0.154	0.035	4.450	0.000	Accept

*EMOL, effectiveness of media online learning; FSMOL, forced shift to media online learning; SI, lack of social interaction; PH, perceived harm; SDAL, perception of maintaining social distancing and law.*

In R square, the researcher used Smart-PLS 3.0 as a tool. Sarstedt et al. (2020) improved the model by including an interaction term. Table 7 shows that the effectiveness of media online learning has been explained up to 70%, while forced shift to media online learning has been explained up to 30% in the structural model.

**TABLE 7:** Assessment of R^2^.

	R square	R square adjusted
EMOL	0.719	0.713
FSMOL	0.324	0.315

*EMOL, effectiveness of media online learning; FSMOL, forced shift to media online learning.*

## Discussion

This section will elaborate on the study’s findings and contribution, as well as describe the effectiveness of online learning with the mediating role of the forced shift to media online learning and the moderating role of student engagement, which will help to predict students’ evaluations during the pandemic. Nevertheless, links have been scientifically investigated both directly and indirectly via intervening factors such as mediating and moderating variables utilizing social learning theory. The results of the analysis will aid in answering the research questions. Each research question on the examination is accompanied by a corresponding hypothesis.

According to the first hypothesis of this study, which is “*Perception of maintaining social distancing and law has a significant effect on the effectiveness of media online learning*,” the findings of this study demonstrate that the perception of maintaining social distancing and law has a large but positive effect on students’ effectiveness of media online learning studying in different universities in China to investigate the relationship between perception of maintaining social distancing and law and students effectiveness of online learning. According to the findings of this study, when students perceive more maintaining social distancing and law within the institution, they are much more effective in online learning.

According to the second hypothesis of this study, which is “*Lack of social interaction has a significant effect on the effectiveness of media online learning*,” the findings of this study demonstrate that lack of social interaction has a significant but positive effect on students’ effectiveness of media online learning studying in different universities in China to investigate the relationship between lack of social interaction and students’ effectiveness of media online learning. According to the findings of this study, when students perceive more lack of social interaction within the institution, they are much more effective in media online learning.

According to the third hypothesis of this study, which is “*Perceived harm has a significant effect on the effectiveness of media online learning*,” the findings of this study demonstrate that perceived harm has a significant but negative effect on students’ effectiveness of media online learning studying in different universities in China to investigate the relationship between perceived harm and students’ effectiveness of media online learning. According to the findings of this study, when students perceive less harm within the institution, they are much less effective in media online learning.

According to the fourth hypothesis of this study, which is “*Forced shift to media online learning has a significant effect on the effectiveness of media online learning*,” the findings of this study demonstrate that forced shift to media online learning has a significant but positive effect on students’ effectiveness of media online learning studying in different universities in China to investigate the relationship between forced shift to media online learning and students effectiveness of media online learning. According to the findings of this study, when students perceive a more forced shift to media online learning within the institution, they are much more effective in media online learning.

According to the fifth hypothesis of this study, which is “*Forced shift to media online learning mediates the relationship between perception of maintaining social distancing and law, lack of social interaction, perceived harm, and effectiveness of media online learning*,” the results of this research show that forced shift to media online learning mediates the association between perceived harm and students’ effectiveness of media online learning. This means that the direct and indirect effects of perceived harm, together with the mediating effect of the forced shift to media online learning, help to retain the student effectiveness in different universities in China.

According to the sixth hypothesis of this study, which is “*Students’ engagement moderates the relationship between forced shift to media online learning and the effectiveness of media online learning*,” the findings of the study show that student engagement acts as a moderator in the association between forced shift to media online learning and students’ effectiveness of media online learning. In other terms, student engagement has a considerable moderating effect on the connection between forced shift to media online learning and students’ effectiveness of media online learning. In practice, students in different universities in China face high student effectiveness due to the fear of pandemic.

## Conclusion

Media online learning is a viable alternative to traditional teaching techniques, and public institutions have shifted rapidly to this environment in the aftermath of the pandemic. The move was unforeseen and unplanned, yet the education had no deterrent effect. This research assessed the effectiveness of media-based online learning during an epidemic when students feel isolated, lonely, and fearful of social distance. According to this research, a lack of social connection is one of the obstacles to effective distance courses; thus, teachers should ensure that their classrooms are engaging. This not only increases the efficiency of media-based online learning, but it also reduces the anxiety associated with feelings of isolation during the lockdown. In contrast, the moderating influence of student participation lowers the efficacy of media online learning. The findings provided substantial evidence for the favorable impacts of the forced shift to media online learning on perceptions of social distance and law, absence of social connection, and perceived damage, all of which enhanced the efficacy of media online learning as a consequence of students’ participation. Additionally, students should be made aware of the hazards of the pandemic in social contexts outside of campus.

### Theoretical Implications

This study has some theoretical implications. First, this study has added value to the literature by investigating the impact of the effectiveness of media online learning on lack of social interaction, perception of maintaining social distancing and law, and perceived harm, with the mediating role of the forced shift to media online learning and by incorporating the role of student engagement as a moderator solely from the perspective of China. The social learning theory was the first to demonstrate it. The social learning theory (SLT) is defined as “how both environmental and cognitive factors interact to influence human learning and behavior” (Deeming and Johnson, 2019). Second, prior literature on SLT theory argued that “perception of maintaining social distancing and law, lack of social interaction, and perceived harm” could be considered as a major input in this process. Hence, the use of these sets, such as “perception of maintaining social distancing and law, lack of social interaction, and perceived harm” by the different universities in China will overcome the problem of the effectiveness of media online learning through SLT. Previous studies have highlighted several factors affecting the effectiveness of media online learning, such as students’ digital literacy, devices, and time spent on online learning, as well as digital literacy and its impacts on parents, teachers, and students. Finally, the results are a great place to start talking about whether the effectiveness of media online learning should be tied more closely to theories and studies.

### Practical Implications

Furthermore, this research provides policymakers, practitioners, and managers with relevant information in a variety of ways. The findings are useful for national authorities and educational institutions to understand students’ perceptions of maintaining social distance and the law. Students believe that keeping a social distance is beneficial to their health and wellbeing, and institution-reopening decisions should be made based on learners’ overall security. To compensate for the lack of education and learning time caused by the pandemic that has affected many all over the world, many educational institutions have introduced online training and educational methods. Institutions have no choice but to adjust to the new standard to mitigate the damage and/or take advantage of the new conditions as a result of the pandemic’s imposed quick shift to media learning. As a result, managers and policymakers should develop a fair policy for implementing media online learning that takes into account students’ behaviors, attitudes, and perceptions. To alleviate students’ anxieties and frustrations about being away from their families and friends and their classrooms, it is essential to maintain a high level of motivation in virtual classrooms until schools and colleges in most countries reopen. Student tiredness and dissatisfaction might be exacerbated by media online learning, which does not encourage students to participate privately. In contrast, events that foster social contacts and private talks among students should be encouraged. In a conclusion, a researcher may claim that students’ success in media online learning is linked to the use of forced shifting to online learning.

### Limitations and Recommendations

Measuring the function of social distance in the new normal, the efficacy of forced shift to media online learning, and the moderating influence of student participation throughout the pandemic have some constraints. First, even though the purpose of this research was to assess the cross-sectional method, the data were obtained at a single point in time. Future researchers may benefit from a longitudinal study design. Second, even if this study reviews and adds a new methodological approach to the literature on university education business, there are a number of limitations to consider. This study focused only on students in Chinese universities. Future researchers should widen their coverage to include additional cities since this study was only designed to reflect the perspectives of Chinese university students about the usefulness of media online learning in their homes. The emphasis of research should be on the cities in which the severity of the issue may have a negative impact on the positive attitudes of students. Third, this study used stratified sampling; however, convenient sampling could give better results for this study. Finally, this outbreak has shown our society that until vaccinations are developed, the catastrophe will continue for some time. Future research could concentrate on gaining a better understanding of the numerous activities that can promote interaction in an online context. Teachers must go outside the boundaries to create themes that allow students to connect while learning. The study will aid instructors and educational institutions because it will allow them to define techniques to improve social interaction in online learning and investigate ways to improve the impact of media in online learning.

## Data Availability Statement

The original contributions presented in this study are included in the article/supplementary material, further inquiries can be directed to the corresponding author.

## Ethics Statement

The studies involving human participants were reviewed and approved by the Hunan International Economics University, China. The patients/participants provided their written informed consent to participate in this study. The study was conducted in accordance with the Declaration of Helsinki.

## Author Contributions

QL: conceptualization and writing the draft. SM: data collection and supervision. Both authors contributed to the article and approved the submitted version.

## Conflict of Interest

The authors declare that the research was conducted in the absence of any commercial or financial relationships that could be construed as a potential conflict of interest.

## Publisher’s Note

All claims expressed in this article are solely those of the authors and do not necessarily represent those of their affiliated organizations, or those of the publisher, the editors and the reviewers. Any product that may be evaluated in this article, or claim that may be made by its manufacturer, is not guaranteed or endorsed by the publisher.
